# Interference with Processing Negative Stimuli in Problematic Internet Users: Preliminary Evidence from an Emotional Stroop Task

**DOI:** 10.3390/jcm7070177

**Published:** 2018-07-18

**Authors:** Adriano Schimmenti, Vladan Starcevic, Alessia M. Gervasi, Jory Deleuze, Joël Billieux

**Affiliations:** 1 Faculty of Human and Social Sciences, UKE-Kore University of Enna, 94100 Enna, Italy; 2 Discipline of Psychiatry, Sydney Medical School-Nepean, University of Sydney, Sydney, NSW 2751, Australia; vladan.starcevic@sydney.edu.au; 3 Department of Human Sciences, University of Verona, 37138 Verona, Italy; alessiamaria.gervasi@univr.it; 4 Laboratory for Experimental Psychopathology, Psychological Science Research Institute, Université Catholique de Louvain, 1348 Louvain-la-Neuve, Belgium; jory.deleuze@uclouvain.be; 5 Addictive and Compulsive Behaviours Lab, Institute for Health and Behaviour, University of Luxembourg, Esch-sur-Alzette 4375, Luxembourg; joel.billieux@uni.lu

**Keywords:** Internet addiction, emotional Stroop, negative emotions, behavioral addictions, problematic Internet use

## Abstract

Although it has been proposed that problematic Internet use (PIU) may represent a dysfunctional coping strategy in response to negative emotional states, there is a lack of experimental studies that directly test how individuals with PIU process emotional stimuli. In this study, we used an emotional Stroop task to examine the implicit bias toward positive and negative words in a sample of 100 individuals (54 females) who also completed questionnaires assessing PIU and current affect states. A significant interaction was observed between PIU and emotional Stroop effects (ESEs), with participants who displayed prominent PIU symptoms showing higher ESEs for negative words compared to other participants. No significant differences were found on the ESEs for positive words among participants. These findings suggest that PIU may be linked to a specific emotional interference with processing negative stimuli, thus supporting the view that PIU is a dysfunctional strategy to cope with negative affect. A potential treatment implication for individuals with PIU includes a need to enhance the capacity to process and regulate negative feelings.

## 1. Introduction

In recent years, the Internet has modified our approach to work, relationships, and leisure time activities [1]. However, there is a darker side to this “digital revolution” [2], as reflected by the excessive or otherwise problematic use of the Internet itself [3]. This problem is not new to researchers and clinicians: two decades ago, Young [4] and Griffiths [5] described cases presenting with “Internet addiction”, a concept that has since been extensively studied (see [6] for a review). Empirical research and theoretical considerations have resulted in an ongoing and intense debate among scholars as to how excessive and repetitive use of various Internet applications should be conceptualized. While some authors regard such use as akin to addictive disorders [7], others see it as a maladaptive coping strategy related to adjustment problems [8,9] and/or as a manifestation of an underlying psychopathology [10,11,12].

Different conceptualizations of problematic Internet use (PIU) have various etiological, diagnostic, and treatment implications. In this context, it is imperative to deepen our understanding of the processes involved in the onset and maintenance of PIU. An experimental psychopathology approach might help to disentangle the effects of hypothesized predictors on the development of PIU. In this respect, negative feelings such as loneliness, anxiety, and depressive mood have been consistently linked to PIU [12,13,14], but cognitive processing of these feelings has been relatively neglected in research.

In the current study, we used a computerized emotional Stroop task [15,16] to assess the implicit bias toward positive and negative stimuli in a sample of individuals who completed questionnaires on Internet addiction symptoms as a measure of PIU and on current affect states. Only a few studies have used the Stroop task within the Internet addiction or PIU framework. For example, Dong et al. [17] reported an impaired inhibition control among individuals with Internet addiction performing the classic Stroop task. Metcalf and Pammer [18] further reported that individuals who displayed severe symptoms of addiction to a massively multiplayer online role-playing game (MMORPG) had significantly longer reaction times to negative words and MMORPG-related words than neutral words, whereas such bias was not present among the participants in their study who did not play the MMORPG or were playing MMORPG without displaying addictive-like behaviors. Similar findings were reported by Jeromin et al. [19], who found attentional biases toward computer-related stimuli in excessive Internet gamers by using a modified Stroop version: excessive gamers showed longer reaction times to computer-related words compared to neutral words. However, Jeronim and colleagues were not able to replicate these findings in another study [20].

In line with a compensatory perspective on PIU that regards excessive use of the Internet as a coping strategy to deal with internal and interpersonal difficulties [10,21,22], we hypothesized that compared to individuals without PIU, those with prominent PIU symptoms would exhibit a heightened cognitive interference following the presentation of negative emotional words.

## 2. Method

### 2.1. Participants

The study sample includes 100 consecutively recruited Caucasian right-handed adults (54 females). Participants ranged in age from 18 to 42 years old (*M* = 24.41, *SD* = 5.28). The mean number of years of education was 13.55 (*SD* = 1.89). Male participants were slightly older than females (*M* = 25.57, *SD* = 6.03, and *M* = 23.04, *SD* = 3.89, respectively; *t*_(98)_ = 2.45, *p* = 0.016) and less educated (*M* = 13.15, *SD* = 1.96, and *M* = 14.02, *SD* = 1.72, respectively; *t*_(98)_ = 2.36, *p* = 0.021).

### 2.2. Procedure

After receiving approval for the study from the Internal Review Board for psychological research of the first author’s university, participants were recruited through public announcements placed in local stores of the city of Enna (Italy). People who contacted the research office were asked to participate in a computerized study on emotions and Internet use, and the study was briefly described to them. Those who agreed to participate signed the informed consent and were administered a questionnaire on socio-demographic variables, self-report measures for assessing affect states and PIU, and a computerized emotional Stroop task. A senior psychologist supervised the entire testing process. Participants did not receive any compensation for their involvement in the study.

### 2.3. Measures

#### 2.3.1. Internet Addiction Test (IAT)

The IAT [4] is a 20-item self-report questionnaire that was developed to quantify the severity of “Internet addiction”, and it was used in the present study as an instrument for assessing PIU. The IAT assesses the extent of an inability to control Internet use, preoccupation with the Internet, hiding of Internet use or lying about it, and continued use of the Internet despite its negative consequences. The items are rated on a 5-point Likert scale ranging from 1 (never) to 5 (always), with IAT scores ranging from 20 to 100 and higher scores indicating greater levels of PIU. A cut-off value of 50 is used to identify people with PIU [4]. The IAT includes questions such as “How often do you fear that life without the Internet would be boring, empty, and joyless?” The instrument has been validated in many countries, including Italy [23], and it is still the most frequently used scale to measure Internet-related problems [6]. It has demonstrated good internal consistency and adequate validity in many studies [24]. In the present study, Cronbach’s alpha for IAT was 0.89.

#### 2.3.2. Positive and Negative Affect Schedule-Brief Version (PANAS)

The PANAS [25] comprises two scales, one measuring positive affect and the other measuring negative affect. Participants are required to respond to a 20-item questionnaire (10 items assessing positive affect and 10 items assessing negative affect) using a 5-point scale that ranges from 1 (very slightly or not at all) to 5 (extremely). In this study, the state version of the PANAS was used (PANAS-S) to control for emotional state of participants just before completing the emotional Stroop task. Scores on the PANAS range from 10 to 50 on each scale, with higher scores on the positive affect scale indicating a more pronounced positive mood (e.g., “enthusiast”) and higher scores on the negative affect scale indicating a more pronounced negative mood (e.g., “nervous”). Reliability and validity of the PANAS were found to be adequate in both non-clinical and clinical samples [26]. The Italian translation of the PANAS has demonstrated good psychometric properties, including good internal consistency, adequate test–retest correlation, and good factorial and construct validity [27]. In the current study, Cronbach’s alpha for the positive affect scale was 0.82, while Cronbach’s alpha for the negative affect scale was 0.90.

#### 2.3.3. Emotional Stroop Task (EST)

The Emotional Stroop Task [15,16,28] has been widely used to assess attentional bias for negative or threatening stimuli. In this study, participants were asked to identify the ink color of a word presented on a computer screen, while the word’s meaning was either neutral or emotional (negative or positive); we also used colored words as an additional control for Stroop response time. Participants were instructed to press, as quickly and accurately as possible, the button for the color in which the target word was written. The target words were presented at the center of the participants’ visual field in one of the four colors (red, green, blue or yellow). The keyboard keys corresponding to each color (red = a, green = d, blue = j and yellow = l) were displayed in the upper half of the computer screen. Words from the four categories were taken from a previous study [29]: (1) positive emotional words (e.g., joy, love, proud); (2) negative emotional words (e.g., angry, fear, pain); (3) neutral or control words (e.g., chair, pencil, shoe); and (4) colored words (e.g., cyan, brown, pink). Different word categories were presented in blocks of 25 words with a fixation cross between conditions, followed by the instruction that a new block would be presented in 10 seconds. The words were presented with a fixed duration for 3 s, followed by an inter-stimulus interval in which a fixation cross was presented with a mean duration of 0.5 s. The total duration of each block was 95 s. The fixation cross between the blocks was displayed for 5 s and the instruction for a new block for 10 s. Before the experiment, participants practiced the task in a short training session with rows of letters to get used to the response buttons. The total experiment time was around 10 min. To assess attentional bias toward emotional words, it is usually acceptable to calculate the mean reaction time to name the colors of emotional words and subtract from it the mean reaction time to name the colors of neutral words [30]. The difference in response time usually defines the emotional Stroop effect or ESE [31]. We addressed the common problem of errors in responses to words by calculating the mean response time for each block of word stimuli, and then weighting the mean for the number of errors in the block using the following equation:

Corrected response time = Average block response time in milliseconds × (25 / number of correct block responses).

Since errors in responses are meaningful in the context of word-based emotional Stroop task and as they can reflect the interference of word arousal on task performance [32], this method allowed us to apply weighted penalties for errors that did not alter the reaction times of participants to the task, and thus the estimation of the ESEs.

The experiment was implemented with the program Inquisit 4.0 on an Intel computer with Core 2 Duo T6600 2.20 GHz, 4 (2 × 2) GB RAM 500 GB HDD, 39.4 cm/15.5″ 1366 × 768 glare, ATI Radeon HD 4570 512MB.

### 2.4. Statistical Analyses

Descriptive statistics were computed for all variables. After calculating corrected ESEs for positive and negative words, we divided the participants into two groups, based on the commonly used IAT cut-off value of 50 for identifying people at risk for Internet addiction (i.e., with moderate to severe Internet addiction symptoms) [4] and on several studies that effectively used this score to discriminate between individuals with and without PIU (see [33,34,35,36] for Italian studies that used this cut-off value). These two groups identified participants “without PIU” (*n* = 91, with normally distributed IAT scores ranging from 20 to 49) and “with PIU” (*n* = 9, with IAT scores ranging from 53 to 66). A multivariate analysis of covariance (MANCOVA) was then used to test differences between participants with and without PIU in terms of the study variables, considering socio-demographic variables (gender, age, years of education) as covariates. Finally, we conducted a MANOVA with repeated measures to test for the effect of the PIU groups on ESEs, using positive and negative ESEs as within-subject factors, gender and PIU as between-subject factors and age and years of education of participants, their PANAS-S scores and corrected response time for colored word as covariates.

## 3. Results

Nine (9%) participants reported scores of 50 or above on the IAT, indicating PIU in this study. Four (44%) out of these nine participants were males (Fisher’s exact test *p* = 0.73). Descriptive statistics are presented in Table 1 for the full sample and for the two groups based on the PIU classifications.

A MANCOVA with PIU classification as a factor and socio-demographic variables (gender, age, and years of education) as covariates showed a significant intercept (*F*_(10,85)_ = 7.86, *p* < 0.001, partial *η*^2^ = 0.480) and a multivariate effect of PIU on the study variables (PANAS-S scores, number of errors, and corrected response time for each block: *F*_(10,85)_ = 2.12, *p* = 0.031, partial *η*^2^ = 0.199), whereas the socio-demographic variables were not significant in the model. Univariate analyses further showed that a greater number of years of education increased response time to positive words (*F*_(1,94)_ = 4.56, *p* = 0.035; *B* = 40.79, *SE* = 19.11, partial *η*^2^ = 0.046) and colored words (*F*_(1,94)_ = 4.26, *p* = 0.042; *B* = 48.69, *SE* = 23,590, partial *η*^2^ = 0.043). These analyses also demonstrated that PIU significantly decreased positive affect scores (*F*_(1,94)_ = 5.48, *p* = 0.021, partial *η*^2^ = 0.055) and increased negative affect scores (*F*_(1,94)_ = 4.13, *p* = 0.045, partial *η*^2^ = 0.042) on the PANAS-S, while having a surprisingly significant and negative effect on the number of errors to positive words in the Stroop task (*F*_(1,94)_ = 4.51, *p* = 0.036, partial *η*^2^ = 0.046). No other significant model effects were found in this analysis.

**Figure 1:**
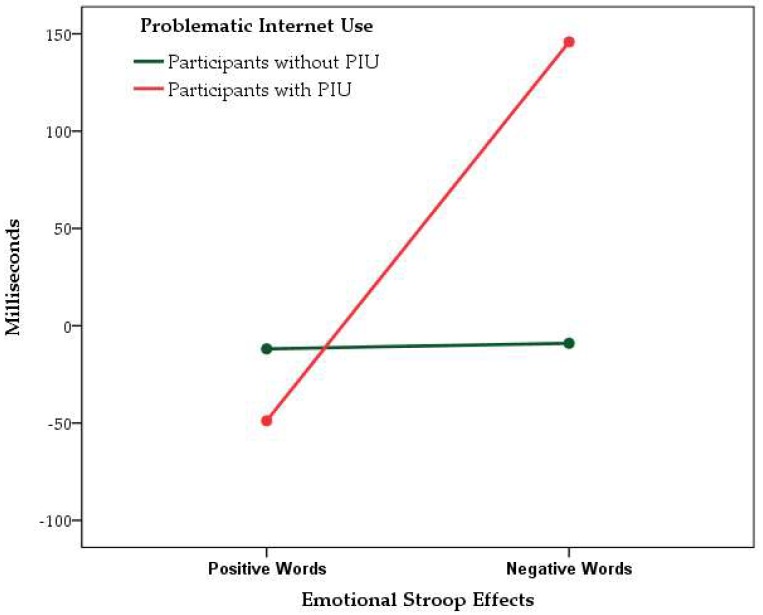
Estimated marginal means for emotional Stroop effects. Note: problematic Internet use = Internet addiction test score of 50 or above. Covariates appearing in the model are evaluated at the following values: age (in years) = 24.41, education (in years) = 13.55, PANAS-Positive States scale = 36.23, PANAS-Negative States scale = 17.17, colored words (in milliseconds) = 1170.55.

Finally, we applied a MANOVA with repeated measure using the Greenhouse–Geisser’s procedure for correcting the degrees of freedom of its *F*-distribution, as Mauchly’s test of sphericity was significant (*p* < 0.05). In line with our hypothesis, the within-subject effect of the interaction between ESEs and PIU was significant (*F*_(1.00,91.00)_ = 4.937, *p* = 0.029, partial *η*^2^ = 0.051), whereas all other within-subject effects were not significant (0.01 < *F* < 1.94, all *p* = ns), as well as all between-subject effects (0.00 < *F* < 0.61, all *p* = ns). Analysis of parameter estimates showed that participants with PIU did not differ from other participants in positive word ESE (*B* = 34.77, *SE* = 116.07; *t* = 0.30, *p* = 0.765, *η*^2^ = 0.001), but they showed increased negative word ESE (*B* = 262.44, *SE* = 106.27; *t* = 2.47, *p* = 0.015, *η*^2^ = 0.063). The estimated marginal means for positive and negative word ESEs in participants with PIU and other participants are displayed in Figure 1.

## 4. Discussion

Although this is not the first time that Stroop effects on emotional words have been tested in relation to PIU, our unique study contributes to the field of PIU research by showing that people who display prominent Internet addiction symptoms may process negative stimuli differently from those without such symptoms. Participants with PIU displayed decreased positive affect and increased negative affect at the PANAS in our study, indicating that they have more prominent negative emotional states in comparison to other participants. Moreover, the increased emotional Stroop effects in participants with PIU were only related to words with negative emotional valence.

As several studies have shown a significant association between PIU and the presence of internalizing symptoms such as anxiety and depression [8,14,37,38,39,40], lower levels of positive affect and higher levels of negative affect in participants with PIU were expected. However, an unexpected and somewhat counterintuitive result was also observed, because participants with PIU correctly responded to a greater number of positive emotional words.

The results of the MANOVA with repeated measures might help to interpret these apparently contradictory findings because they suggest that people with PIU may have a *specific* difficulty with cognitive processing of the negative emotional stimuli. As different covariates were controlled for in this study, including current affect states, it is likely that the increased Stroop effects for negative emotional words among participants with PIU reflected a slower processing of negative stimuli and/or interference of such stimuli with the task. This is in contrast to participants without PIU who processed negative emotional words as fast as positive emotional words.

Therefore, our findings are in agreement with previous research showing compromised cognitive control over negative stimuli in problematic Internet users. For example, Lee and colleagues [41] showed that adolescents with PIU who completed an emotional task were more distracted than those without PIU by being presented with angry faces. This may suggest that attentional bias toward negative stimuli in people with PIU disrupts goal-directed attention. Furthermore, another study [42] indicated that people with PIU might have cognitive deficits associated with an adjustment to negative emotions.

Although findings of our study and other research suggest that people with PIU often experience a specific difficulty processing negative emotions, it is uncertain whether such a difficulty is antecedent to PIU or whether it is caused or enhanced by PIU. As we controlled for current affect states in our study, it appears that problems with processing negative emotions preceded PIU, but the opposite direction of causality cannot be excluded.

Considering the fact that individuals with PIU correctly responded to a greater number of positive emotional words and showed increased emotional Stroop effects for negative words compared to other participants in our study, we can speculate that PIU develops in two stages. First, some people may be attracted by one or more Internet applications that temporarily allow them to fulfill a need to experience positive emotions such as joy, excitement or relaxation or to cope with emotional difficulties (e.g., problems in close relationships). In the second stage, a number of these people develop an addiction-like behavior in relation to these specific Internet applications for the same reason that they were initially attracted to them. However, this addiction-like behavior may further exacerbate their original emotional problems and increase their negative affect, thus creating a vicious cycle. These speculations are in line with the I-PACE model (interaction of person, affect, cognition and execution) [43], which postulated close links between emotional and personality features on one hand and excessive and specific online behaviors on the other. Regardless of the theoretical stance, findings of the present study have clinical implications in terms of a need to foster the capacity to modulate and tolerate negative feelings in individuals with PIU.

As with all research, this study comes with a number of limitations. They include the small number of participants with PIU, reliance on self-reported measures to assess PIU and negative affect and participation in the study of individuals from the community who reported prominent symptoms of PIU instead of people with *bona fide* PIU or those from a clinical sample. This limits the generalizability of our findings. Moreover, our findings should not lead researchers and clinicians to underestimate the potential role of impaired response inhibition and abnormal sensitivity to reward and punishment in PIU. Altered functioning in brain regions such as the posterior cingulate cortex and the prefrontal and parietal regions has been observed both in PIU (see for reviews [44,45]) and substance use disorders, suggesting that in some cases PIU may be related to a generalized impairment in emotional processing. Therefore, further studies of people who seek professional help for PIU, using interview-based instruments and experimental designs and taking into account the interaction between emotional processing and executive functions are warranted to advance research.

## 5. Conclusions

Notwithstanding the limitations of the study, its findings represent a novel and significant contribution to the literature. The study demonstrates that cognitive processing of negative emotions may be specifically altered in people with PIU. This not only allows for a better understanding of PIU and its emotional and cognitive correlates, but can also inform tailored treatment of individuals for whom PIU has already generated significant impairments. In fact, some people with PIU may be able to better control their excessive Internet-related behaviors if they also become more capable of processing their negative emotions. It would then be important to link such emotions with the personal and environmental factors that increase susceptibility to use the Internet excessively as a way of satisfying psychological needs or as a dysfunctional coping strategy. Finally, clinical interventions aimed at improving ability to process and regulate negative feelings may be important for both resolution of PIU and relapse prevention, as well as improving the quality of life of people suffering from PIU.

## Figures and Tables

**Table 1:** Descriptive statistics.

	Full Sample (*n* = 100)		No PIU (*n* = 91)	PIU (*n* = 9)
	M (SD)	Range	M (SD)	M (SD)
Age	24.41 (5.28)	18–42	24.68 (5.42)	21.67 (2.50)
Education (years)	13.55 (1.89)	8–18	13.60 (1.98)	13.00 (0.00)
Internet Addiction Test	35.42 (9.93)	20–66	33.42 (7.87)	55.67 (4.15)
PANAS—Positive states	36.23 (5.80)	20–49	36.65 (5.50)	32.00 (7.33)
PANAS—Negative states	17.17 (7.45)	10–40	16.71 (7.28)	21.78 (8.16)
Positive word score	23.98 (1.11)	21–25	23.90 (1.14)	24.78 (0.44)
Neutral word score	24.06 (1.26)	20–25	24.04 (1.29)	24.22 (0.97)
Negative word score	24.07 (1.32)	20–25	24.07 (1.33)	24.11 (1.37)
Colored word score	24.13 (1.37)	17–25	24.13 (1.42)	24.22 (0.83)
Positive words (ms)	1104.65 (341.95)	613.08–2310.17	1108.67 (337.33)	1064.03 (405.96)
Neutral words(ms)	1118.42 (350.71)	630.46–2176.09	1114.95 (338.51)	1153.57 (481.12)
Negative words (ms)	1127.76 (376.03)	619.21–2618.64	1111.73 (352.40)	1298.89 (565.92)
Color words (ms)	1170.54 (429.19)	611.50–2529.52	1165.12 (411.73)	1225.43 (607.20)
ESE positive words (ms)	−13.77 (232.45)	−1105.09–1150.29	−6.28 (239.54)	−89.53 (126.80)
ESE negative words (ms)	9.34 (217.56)	−1010.57–764.21	−3.22 (218.63)	136.32 (167.61)

Note: PIU–problematic Internet use; PANAS—Positive Affect Negative Affect Schedule; ESE—emotional Stroop effect.
